# 3D shear wave velocity model of the crust and uppermost mantle beneath the Tyrrhenian basin and margins

**DOI:** 10.1038/s41598-019-40510-z

**Published:** 2019-03-05

**Authors:** D. Manu-Marfo, A. Aoudia, S. Pachhai, R. Kherchouche

**Affiliations:** 1 The Abdus Salam International Center for Theoretical Physics, Trieste, Italy; 2 University of Trieste, Trieste, Italy; 3 Bullard Laboratories, Department of Earth Sciences, University of Cambridge, Cambridge, UK; 4 Now at Institute of Geophysics and Planetary Physics, Scripps Institution of Oceanography, University of California, San Diego, California, USA; 5 Université des Sciences et de la Technologie Houari Boumediene, Algiers, Algeria; 6 Present Address: Centre de Recherche en Astronomie, Astrophysique et Géophysique, Algiers, Algeria

**Keywords:** Geodynamics, Volcanology, Seismology

## Abstract

The Tyrrhenian basin serves as a natural laboratory for back-arc basin studies in the Mediterranean region. Yet, little is known about the crust-uppermost mantle structure beneath the basin and its margins. Here, we present a new 3D shear-wave velocity model and Moho topography map for the Tyrrhenian basin and its margins using ambient noise cross-correlations. We apply a self-parameterized Bayesian inversion of Rayleigh group and phase velocity dispersions to estimate the lateral variation of shear velocity and its uncertainty as a function of depth (down to 100 km). Results reveal the presence of a broad low velocity zone between 40 and 80 km depth affecting much of the Tyrrhenian basin’s uppermost mantle structure and its extension mimics the paleogeographic reconstruction of the Calabrian arc in time. We interpret the low-velocity structure as the possible source of Mid-Ocean Ridge Basalts- and Ocean Island Basalts- type magmatic rocks found in the southern Tyrrhenian basin. At crustal depths, our results support an exhumed mantle basement rather than an oceanic basement below the Vavilov basin. The 3D crust-uppermost mantle structure supports a present-day geodynamics with a predominant Africa-Eurasia convergence.

## Introduction

The Tyrrhenian basin is a back-arc basin in the Mediterranean region which opened in relation to the retreating Adriatic-Ionian slab in the geodynamic context of the African and Eurasian plates convergence^1–3^. Being the youngest basin in the Central Mediterranean region, the Tyrrhenian is considered as a reference natural laboratory for investigating the geodynamics of back-arc basins^4–8^. However, the crust and upper mantle velocity structure is poorly understood beneath the basin and adjacent margins. The distribution of seismic stations on land makes it seismically challenging to image the shallow lithosphere below the Tyrrhenian basin, as it inhibits proper illumination of the shallow structures by teleseismic compressional (P) and shear (S) waves^9^. Additionally, teleseismic surface waves mostly lack short periods sensitive to the crust and uppermost mantle structure beneath the basin. Consequently, previous tomographic studies have limited resolution of the shallow lithospheric structure beneath the basin^10–12^.

An alternative method to study the lithosphere, particularly the crust, is active seismological observations. In fact, most of our knowledge about the crust beneath the Tyrrhenian basin stems from such studies. For instance, Moeller *et al*.^8^ have shown that the north Tyrrhenian basin is underlain by a continental crust of about 17 km thick but Vp values of the lower crust are higher than those found for the average continental crust. Again, Prada *et al*.^13^ have found evidence for exhumed mantle rocks beneath the Vavilov basin, contrary to the previous suggestion of an oceanic crust^14,15^. However, the problem with active seismological studies is that it provides very little information about the lithospheric structure beneath the Moho and also yields relatively limited information on the lateral variation of the velocity structure.

In over a decade now, ambient noise tomography has proven to be a valuable tool for imaging crust and lithospheric mantle velocity structure^16,17^. The method has become particularly essential in areas where there exist difficulties in achieving high-resolution images of the lithosphere using data from ‘traditional’ seismic imaging techniques^18,19^. This method has been already applied in the Tyrrhenian area^20–23^, but in the framework of regional studies covering the whole Europe^20–22^ or the Italian peninsula^23^ and therefore does not provide much detailed structure beneath the Tyrrhenian basin.

**Figure 1:**
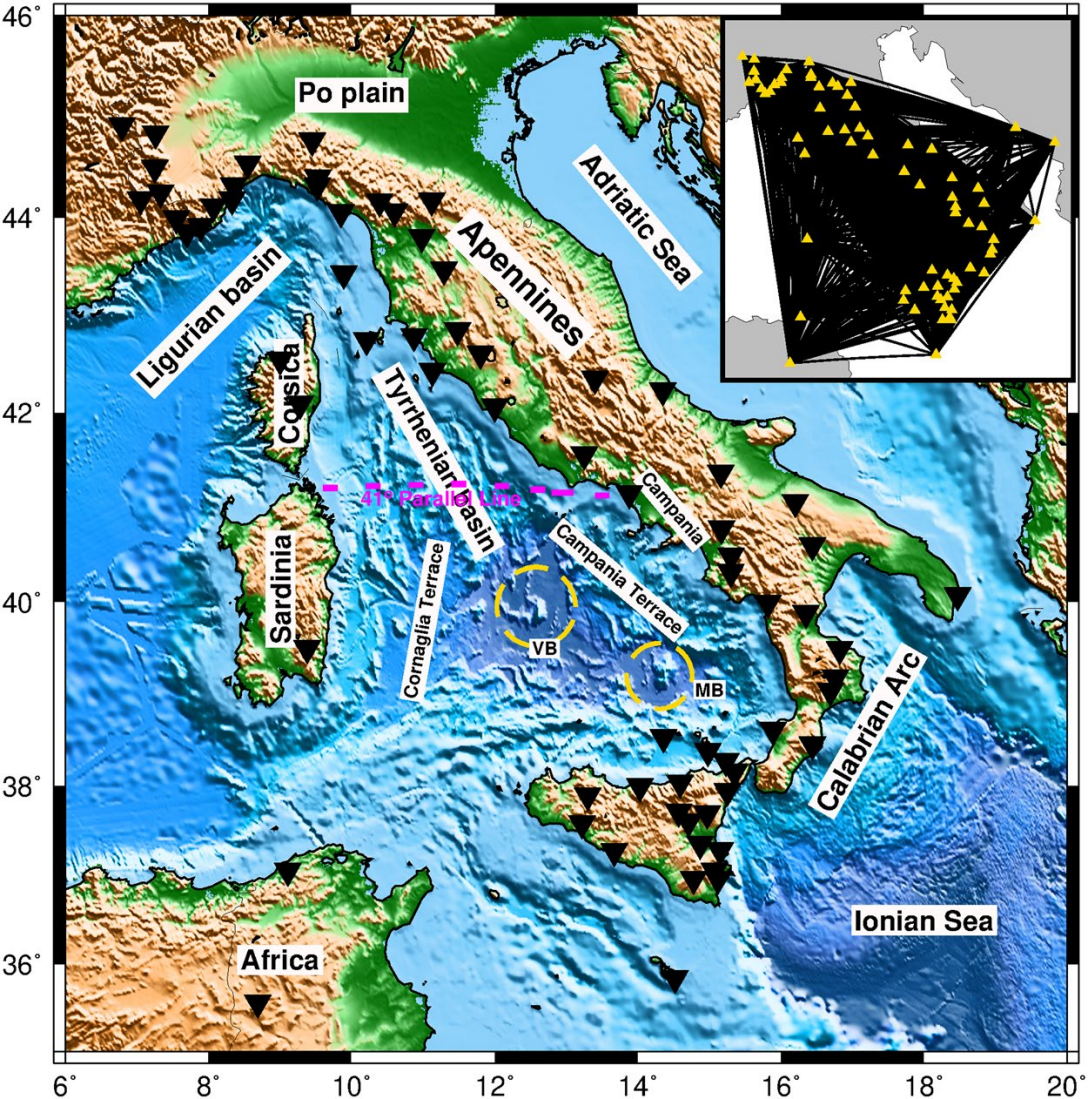
Bathymetric and topographic map of the Tyrrhenian basin and surroundings. Black triangles represent the location of broadband stations used in this study. Inset map shows the ray density with all inter-station paths used in this study. VB = Vavilov basin; MB = Marsili basin.

In this study, we extract interstation Empirical Green’s functions (EGFs, e.g., Supplementary Fig. S1) from the cross-correlation of ambient noise data using a dense network of 73 broadband stations surrounding the Tyrrhenian basin (Fig. 1). This allows for the retrieval of high-quality Rayleigh wave group and phase dispersions, which are inverted to obtain group and phase tomography maps, respectively. Local dispersion curves are then extracted from the tomographic maps. These dispersion curves are traditionally inverted for S-wave velocity applying linearized inversion, which iteratively minimizes the objective function until certain misfit value is achieved^24^. However, such inversion can often lead to a misleading solution if the starting model is not close to the true one and can encounter challenges on the estimation of proper uncertainties, particularly for highly non-linear problems. Here, we implement a highly efficient trans-dimensional Bayesian approach^25,26^ and provide a new 3D shear wave velocity structure beneath the Tyrrhenian basin and its margins (down to 100 km depth) along with the related uncertainties. In this approach, layer properties including the number of layers remain unknown in the inversion and fully constrained by data. To provide efficient sampling and achieve faster convergence, we adapt an interacting Markov chain Monte Carlo Sampling approach in which parameters are allowed to exchange between different chains. We identify new structural features in the Tyrrhenian basin and at the transition with the surrounding Apennines, Calabrian arc, and Sardinia block. We discuss our results in light of the published recent findings in terms of structure, magmatism, and geodynamics.

## Results

### Group and phase tomography maps

**Figure 2:**
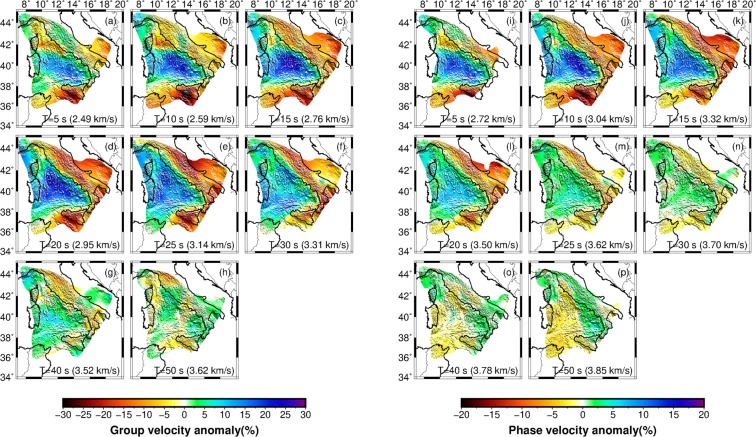
Rayleigh wave tomographic maps. (**a**–**h**) Group velocity and (**i–p**) phase velocity maps at periods of 5, 10, 15, 20, 25, 30, 40 and 50 s. Colours represent the percentile deviation from the average velocity at each period shown at the bottom of each plot. Results are shown only for the resolution length shorter than 150 km (Supplementary Fig. S2).

Figure 2 shows the ambient noise tomography maps for group and phase velocities at different periods (between 5 and 50 s). The tomography maps at short periods (5–10 s, Fig. 2a,b,i,j) show pronounced high-velocity anomalies in the southern Tyrrhenian basin, which likely indicates the presence of a high velocity material at shallow depth. In contrast, the northern Tyrrhenian basin is characterized by low-velocity anomalies which distinguish it from the south, and suggest a different crustal structure compared to the southern Tyrrhenian. A noteworthy observation is that the boundary between the north and south anomalies approximately coincides with the 41° Parallel Line which is generally considered as the divide between the northern and southern Tyrrhenian basin. Observed low-velocity anomalies below the Apennines, Sicily, and the Corsica sedimentary basin are consistent with previous studies^8,23,27^ and are likely related to sedimentary basins.

Velocity maps at 15–30 s periods (Fig. 2c–f,k–n) are substantially sensitive to the crustal thickness and the transition between the crust and uppermost mantle. Consequently, high-velocity anomalies in the Tyrrhenian basin likely correspond to the shoaling of mantle material beneath the basin. The low velocity anomalies coincide with regions having thicker than average crust such as below the Apennines where we observe a continuous low velocity belt mimicking the Apenninic mountain ranges. The long-period group and phase tomography maps (40 and 50 s, Fig. 2g,h,o,p) do not preserve the anomaly contrast at the basin-margin transition as reported in shorter period maps likely due to Rayleigh wave velocities sampling upper mantle materials beneath both regions. Note that the group velocity anomalies are stronger than phase velocity as sensitivity amplitudes are higher for group velocity in comparison to phase velocity (Supplementary Fig. S3).

### Shear velocity structure

We construct a 3D shear-wave velocity model with errors by combining 1D velocity-depth profiles at each node of a 0.5° by 0.5° grid across the Tyrrhenian Sea and its margins. Supplementary Figs S4 and S5 show the one standard deviation from the estimated mean S-wave velocity. The uncertainties are higher near the location of interfaces due to smearing of velocity structure. In Supplementary Fig. S6, we plot the mean S-wave velocities on horizontal sections at different depths. Overall, the uncertainties are approximately 4% of the mean velocity which suggests that the velocity is reasonably well constrained.

There is a variable lateral resolution of our model, due to the variation in path density from the cross-correlation (Supplementary Fig. S2). Lateral resolvable structures are on the order of ~30 km in the short-period part of the model and degrade to ~150 km at longer periods (Supplementary Fig. S2). In general, the structures beneath the portion of the basin parallel to the Italian Peninsula are highly resolved due to a large number of stations used from this area and consequent good path coverage. The best vertical resolution is ~5 km at shallow depth and resolution decreases for deeper structure (Supplementary Fig. S3). Inversion results indicate that our model is reasonably well resolved from the surface down to about 100 km (see Supplementary info for depth resolution test).

**Figure 3:**
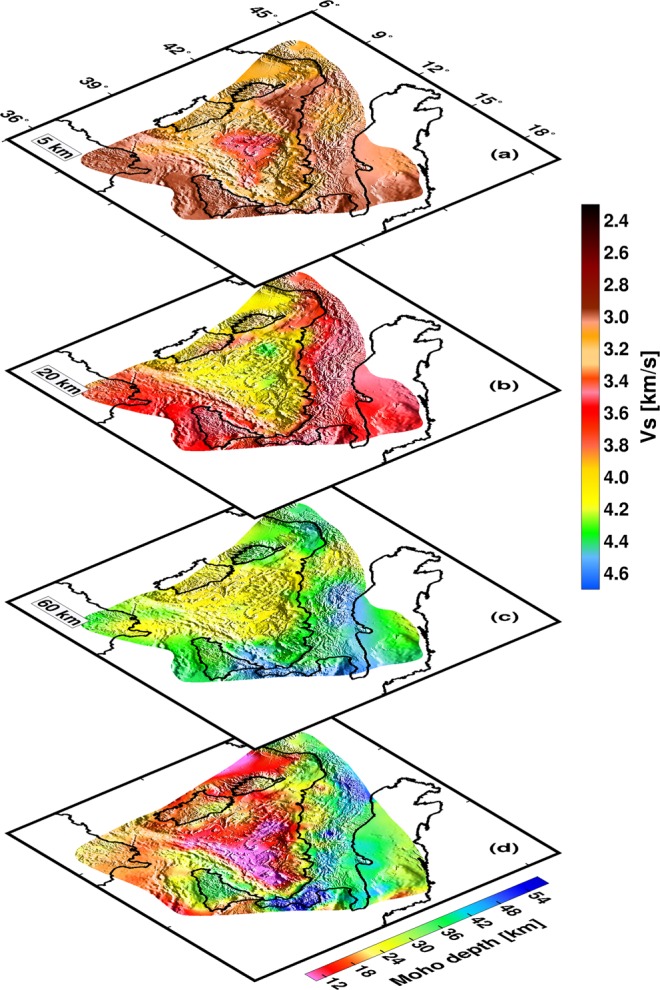
Shear velocity structure in map view and Moho topography map for the study area. (**a**–**c**) Shear velocity structure at 5, 20 and 60 km depth. (**d**) Moho topography map for the Tyrrhenian area.

Figure 3a–c show selected horizontal slices at 5 km, 20 km, and 60 km depth, respectively and Fig. 3d shows the Moho topography (discussed later). In general, the shear velocity model shows similar characteristics as the Rayleigh wave tomography results, yet highlights tectonic and geological features associated with the Tyrrhenian lithosphere. At shallow crustal depths (5 km map, Fig. 3a), low shear velocities are confined to the northern Tyrrhenian basin, Sicily, Calabria, and below the Italian peninsula. The highest velocities occur below the Vavilov basin and are somewhat surrounded by moderate high velocities in the Cornaglia and Campania terraces which extend southeast to reach the Marsili basin.

At 20 km depth (Fig. 3b), we see a clear distinction between the Tyrrhenian basin and its margins, with high velocities delineating the triangular shape of the Tyrrhenian basin. At this depth, we do lose the velocity contrast between the northern and southern Tyrrhenian basin which suggest a likely uniform uppermost mantle structure beneath the basin. Low velocities observed below continental regions can be explained by the presence of crustal material that extends beyond 20 km depth^28^. The 60 km depth map (Fig. 3c) shows a reversal in the velocity pattern, having high velocities beneath the continental regions (Sicily, Calabria, and Italian peninsula) and low velocities below the basin.

**Figure 4:**
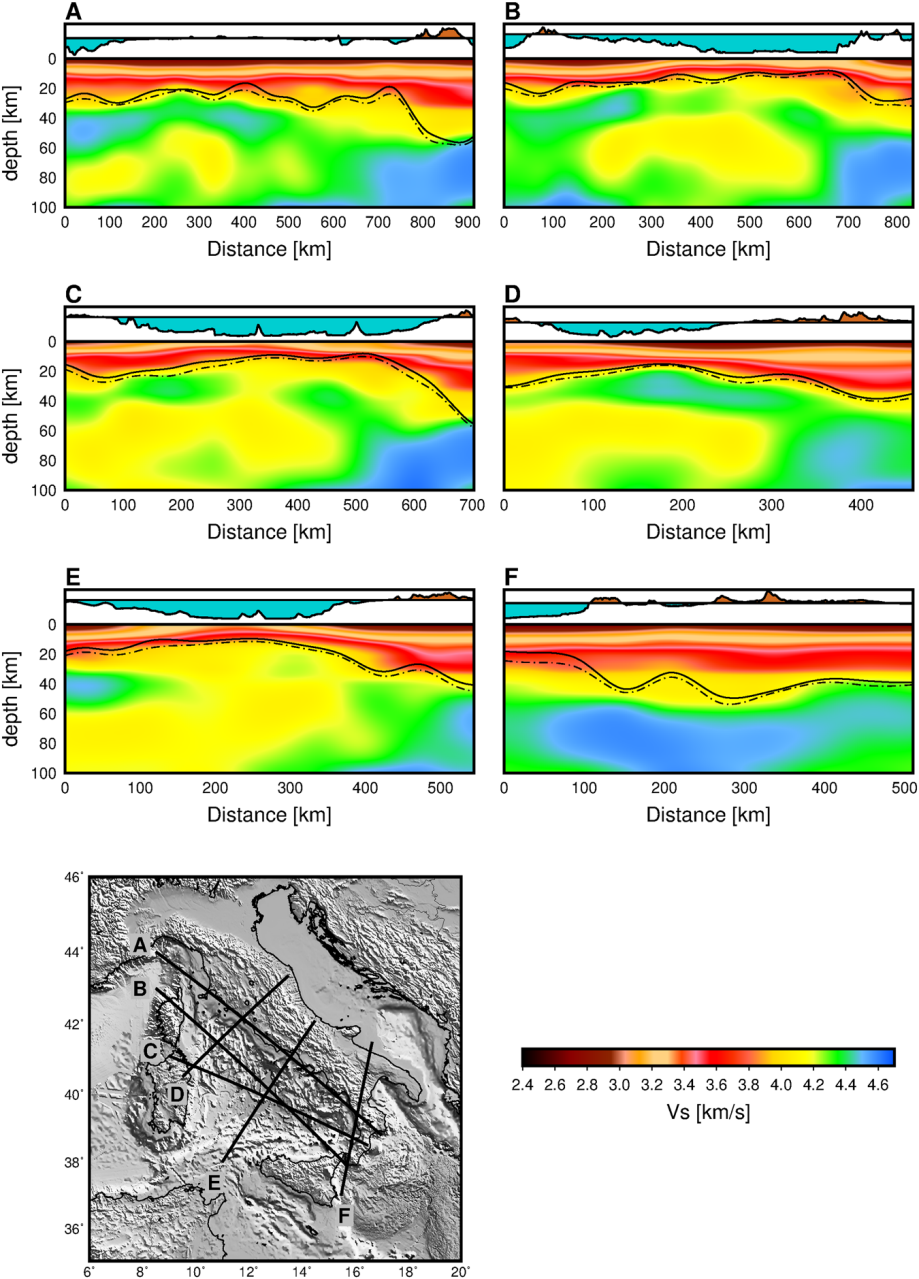
Shear velocity structure along six different cross-sections. The black lines on the cross-sections depict the undulation of the Moho along the profile and the dash-dotted line shows the uncertainties of the Moho depth. The depth scale in the cross sections is exaggerated by a factor of 2 or 3 depending on the length of the profile.

Figure 4 shows the velocity-depth vertical cross sections along six lines. On these cross sections, the black continuous line depicts the Moho topography along the profiles based on the maximum interface probability and the dash-dotted line indicates the depth where the velocity becomes mantle velocity and gradient of velocity is approximately infinite. This can also represent an approximate uncertainties for the determination of Moho. The vertical profiles in Fig. 4 suggest a variable crustal thickness both within the basin and across the margin between the basin and the continental regions. We see that the geometry of the crust is one that thins gradually from about 20 km below the margins to ~10 km beneath the Vavilov and Marsili basins (Profile C and E, Fig. 4). In the northern Tyrrhenian basin, a crust of ~16 km thick is found (Profile D, Fig. 4). The greatest crustal thickness is observed beneath Calabria, extending down to the top of the downgoing high velocity Ionian slab at ~55 km depth (Profiles A and C, Fig. 4). The crustal thicknesses observed in our model are consistent with previously reported results^28–30^.

We see a low velocity zone emerging at about 40 km and extending to ~80 km depth or the base of the model depending on the area crossed by the profile (Profiles B–E, Fig. 4). In profile D crossing the Northern Tyrrhenian, Fig. 4, a high velocity body, likely a remnant of the European lithosphere, sits on top of the low velocity layer. We interpret the high velocity body seen at the start of Profile E (Fig. 4), as the northernmost part of the African lithosphere. The geometry of the mantle lithosphere beneath the Vavilov basin in Profile C and E (Fig. 4), suggests that strong extension that caused the opening of the southern Tyrrhenian may have involved the uppermost mantle, indicated by some form of break-up within the overriding plate. The geometry of the mantle lithosphere along Profile E suggests an ongoing compression between the African block and the already extended mantle lithosphere beneath the Tyrrhenian basin. High upper mantle velocities observed below Calabria are associated with the Ionian slab (Profile A–C and F, Fig. 4).

### Moho topography

In general, surface wave dispersion measurements are sensitive to absolute shear wave velocities but are poor in constraining discontinuities. Hence, for most surface wave studies, the Moho interface is taken as the depth of the 4.2 km/s velocity contour (e.g., ref.^31^). In the Tyrrhenian basin, the complex tectonic history has undoubtedly affected the lithospheric structure and using the 4.2 km/s velocity contour to define the Moho interface in our model produce results that are inconsistent with previous studies. Here, we determine the Moho depth by picking where strong interface probability for a discontinuity occurs at a pertinent shear velocity on the 1-D velocity depth profile (see Supplementary info and Figs S7, S8 and S9).

In Fig. 3d, we present a new Moho topography map for the Tyrrhenian basin and margins. The contour map of the Moho topography is shown in Fig. S10. These maps show strong lateral variation in crustal thickness but very consistent result within the different tectonic provinces of the study area. The Moho is very shallow (about 10–12 km thick) below the southern Tyrrhenian basin. We observe similar shallow Moho depths (~11 km) in the northern Tyrrhenian right above the Vavilov basin (Fig. 3d), in agreement with the results from recent wide-angle seismic reflection studies^32^. The deepest Moho is found below the northern Apennines and Calabria, where the Moho depths exceeds ~50 km. The general characteristics of the Moho thicknesses found here are very consistent with previously reported depths^28–30^. The significant improvement achieved in our model lies in the resolution of the Tyrrhenian basin’s Moho topography which is not well resolved in previous models (e.g., ref.^29^).

## Discussion

**Figure 5:**
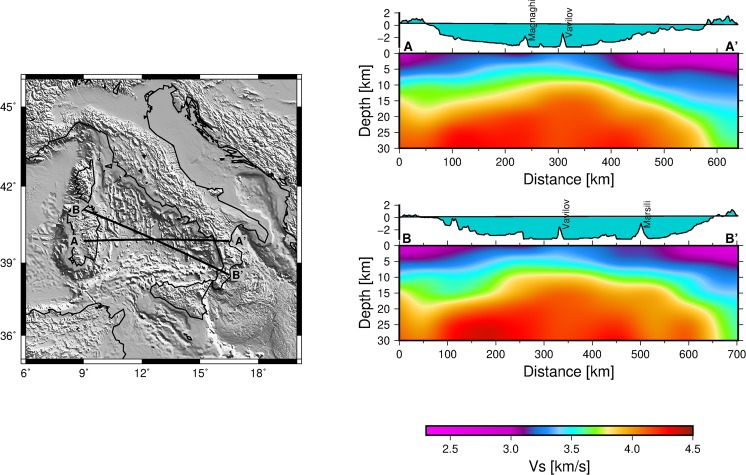
Zoom-in of the crustal velocity model in the Southern Tyrrhenian basin. Section A-A′ is along the active seismic profile by ref.^13^.

It can readily be seen from our images (Figs 2 and 3) that two rheologically different domains underlain the northern and southern Tyrrhenian basin. The continental crust under the northern Tyrrhenian basin^8,33,34^ is characterized by low shear velocities in our model (Fig. 3a). The nature of the crust in the southern Tyrrhenian basin is rather complex. At shallow depths (<10 km), we observe the highest velocities in the central part of the basin, below the Vavilov-Magnaghi basin (Figs 3a and 5). Here, recent active seismological results suggest that strong extension in the southern Tyrrhenian resulted in mantle exhumation beneath the Vavilov basin^7,13,35^ contrary to previous studies suggesting the emplacement of an oceanic crust^14,15^. Our highest velocities at crustal depths (profiles A and B, Fig. 5) occur below the Vavilov basin, which corroborates an exhumed mantle basement below the Vavilov basin^7,13,35^. Integrating our crustal model for the Tyrrhenian basin and margins (Figs 3–5) with recent publications^13,36^, we see a clear delineation of the three basement domains in the southern Tyrrhenian suggested by Prada *et al*.^13^: stretch continental crust (beneath Sardinia and Campania margins), oceanic crust (beneath Cornaglia and Campania terraces) and exhumed mantle basement (below the Vavilov basin). We interpret the structure of the crust under the Marsili basin as oceanic as supported by recent magnetic studies^37,38^.

Remarkably, the contrast in shear velocity structure between the northern Tyrrhenian and the southern Tyrrhenian in our model coincides approximately with the location of the 41° Parallel Line (Fig. 3a, see also the tomography maps at short periods, Fig. 2). The 41° Parallel Line is defined as a regional magnetic and free-air gravity anomaly that is conventionally regarded as separating the northern Tyrrhenian continental structure from the heterogeneous structure in the southern Tyrrhenian^39–42^. The structural significance of 41° Parallel Line in the geodynamic evolution of the basin is still debated^3,40,41,43^, nevertheless, our results provide the first comprehensive seismological evidence for the presence of this lithospheric feature.

In Fig. 3d, we see two expressions of very shallow Moho (~10–12 km) which we infer to be related to the different style of rifting that opened the north and south Tyrrhenian basin^44^. In the north, we interpret the feature of the shallow Moho oriented approximately N-S as the likely expression of the initial eastward retreat of the Adriatic slab that caused the opening of the northern Tyrrhenian basin^3,44^. The second observed shallow Moho feature in Fig. 3d is oriented approximately NW-SE in the south. This feature is clearly the effect of the ESE retreat of the Ionian slab which resulted in the opening of the south Tyrrhenian basin^2,3^.

The most pronounced feature in the upper mantle is the presence of a low velocity zone (LVZ) extending from 40 to 80 km and affecting much of the Tyrrhenian basin upper mantle structure (Fig. 4). Similar velocity decrease has been reported in previous models by Greve *et al*.^11^ and Marone *et al*.^45^. Low velocities in the Tyrrhenian upper mantle likely originate from a number of contributing factors. First, asthenospheric upwelling due to lithospheric extension in the basin may cause decompressional melts which can explain the observed low velocities beneath the basin. Second, the likely presence of a hydrous upper mantle structure below the basin due to past subduction and rollback. Last, the effect of mantle potential temperature on velocity.

Generally, variation in seismic velocity simultaneously depends on temperature, pressure, and composition and the uncertainty in the estimation of these controlling factors makes it difficult to separate their effects on velocity^46^. Nevertheless, seismic velocity anomalies in the upper mantle are generally interpreted in terms of temperature, being that the effect of temperature on seismic velocity is thought to be greater than other controlling factors^10^. Wiens *et al*.^47^ attributed a significant low velocity zone extending from 40 to 100 km depth observed in the shear-velocity structure of four back-arc basins to variations in mantle potential temperature, as the observed velocity decrease show no apparent correlation with variation in water content.

In the Tyrrhenian basin, Greve *et al*.^11^ explained the velocity decrease from 70 to 110 km depth range in terms of variations in water content, alluding that temperature plays a smaller role compared to other back-arc basins^47^. However, considering that the strongest lithospheric extension occurred in the central part, beneath the Vavilov basin^2,3^, we expect the mantle to upwell and decompressional melt to occur similar to a mid-ocean ridge system. The low seismic velocities observed at shallow depth beneath the Tyrrhenian basin is consistent with such a shallow melt zone. We argue that the top of the LVZ at 40 km depth seen in our images (Fig. 4) suggest that in addition to water content variation and temperature, decompressional melts contribute to the observed LVZ under the Tyrrhenian basin.

Volcanism of the Tyrrhenian basin and its margins shows large variations in time and space reflecting the complex tectonic history of the region. Igneous activities in the southern Tyrrhenian basin emplaced a wide variety of magmatic rocks spanning Mid-Ocean Ridge Basalts (MORB)-, Ocean Island Basalts (OIB)-, and Arc- type geochemical signatures^48^. This wide range of magmatism point to a variety of mantle sources and melting processes^48–50^. Below the Vavilov basin, our images (Fig. 4) show low velocities extend from 40 to 80 km depth, well within the estimated range for primary MORB production based on geochemical considerations^51^. We infer that the LVZ below the Vavilov basin feeds the Vavilov–Magnaghi shallow structures and may perhaps be the source of MORB- and OIB- type magmatic rocks found here^15,48^. Although the LVZ is a broad feature beneath the Tyrrhenian basin (see Profile E, Fig. 4), it is still interesting that we see a conduit-like feature below the Vavilov volcanic complex which appears to connect the shallow lithospheric structures to the top of the LVZ at 40 km (Profile C, Fig. 4). Differently from Vavilov, the structure beneath the Marsili seamount is dominated by the high velocity Ionian slab (profile C, Fig. 4), so volcanism here may require a different dynamics which may not be related to the observed velocity decrease in the upper mantle.

The high velocities observed in our images beneath Calabria and extending under the southeast Tyrrhenian basin, point to the Ionian slab subducting below the Calabrian Arc (Profile A–C and D, Fig. 4). Tomography results and intermediate to deep earthquakes define the NW subducting slab down to nearly 400 km depth^9,52^. A Subduction-Transform Edge Propagator (STEP^53^) laterally bounds the distinct edges of the Ionian slab in both the northeast and southwest^54^. In profile F (Fig. 4), we are likely sampling the southwest edge of the Ionian slab, indicated by the clear transition from fast to slower velocities which occur in proximity of the Ionian Fault^54^. This is consistent with multichannel seismic experiment that shows that the Ionian Fault forms part of a complex deformation zone that bounds the southwestern edge of the Calabrian subduction system^54^. In the northeast, we do not see a clear boundary of the Ionian slab beneath the southern Apennines but rather we see a flexing of the fast velocity towards shallow depths (profile F, Fig. 4). The Sicily-Tyrrhenian offshore thrust front^55^ which accommodate the Africa-Europe plate convergence through thrusting type seismicity and structural data^56^ and connects to the Ionian fault system^57^ is seen here as a crustal feature affecting the Moho topography (Fig. 3d).

Overall, the geometry of the crust-upper mantle structure beneath our study area is more in favor of a dominant present-day Africa-Eurasia convergence rather than a slab retreat mechanism that was dominant in the last 30 Myr^55^. The nowadays expression of the slab-retreat mechanism is localised at both lateral edges of the Ionian slab, on its transition to the Sicilian domain in the southwest and southern Apennines domain in the northeast. These two edges delineate the thickest crust in our study area and very well localised beneath Calabria known for its low geodetic strain rates^58^.

## Conclusions

We determined a 3D shear velocity model for the Tyrrhenian basin from the inversion of Rayleigh wave group and phase velocities derived from ambient noise cross-correlations. The inversion results indicate a pronounced low shear velocity layer in the uppermost mantle, between 40 and ~80 km depth, affecting much of the Tyrrhenian basin. We suggest that this low velocity zone is possibly the source of the MORB- and OIB- type magmatic rocks found in the Vavilov basin. The lateral extent of our Moho topography model likely reflects the initial E-W extension in the northern Tyrrhenian and the successive NW-SE extension in the southern Tyrrhenian which resulted in the formation of the basin. The Moho topography mimics the extent of the Sardinia, Campania and Sicily margins as well as the Cornaglia and Campania terraces and Magnaghi-Vavilov basin which is characterized by high velocity and a very shallow Moho likely reflecting mantle unroofing/exhumation. In the Calabrian subduction zone, we find evidence for the Ionian slab edges within the crust and uppermost mantle structure. This is likely to be associated with tearing to the southwest and flexing (or immature tearing) to the northeast, as well as slab narrowing as reported in recent literature. The 3D crust and upper mantle model favors a geodynamic setting where the dominant process is the Africa-Eurasia convergence while slab retreating seems to be less important but localised nowadays beneath Calabria where the thickest crust and highest seismogenesis is reported.

### Data and methods

We use four years continuous data (2010–2013) recorded at 73 broadband stations in Italy, France, Tunisia, Croatia, and Malta (Fig. 1) to compute Rayleigh wave group and phase dispersions curves. The interstation Empirical Green Functions (EGFs) are determined from the noise data by following the processing method of Bensen *et al*.^59^. The day-length vertical component waveforms at individual stations are first demeaned, detrended, corrected for instrumental response and whitened. We minimize the effect of earthquake-generated signals by applying a running-absolute-mean temporal normalization. Finally, the day-length waveforms are cross-correlated between all available station pairs and then stacked to form the EGFs (e.g., Supplementary Fig. S1). We measure dispersion curves from EGFs with a signal-to-noise ratio (SNR) value > 7 and select only cross-correlations with a minimum interstation distance of 100 km. We analyse the EGFs using the multiple filter technique^24^ to measure Rayleigh wave fundamental mode group and phase velocity dispersions from 5 to 50 s period.

The estimation of the velocity structure from the ambient noise data involves a two-step inversion scheme. In the first step, we invert for the group and phase tomography maps on every grid (0.5° by 0.5° size) at different periods (sensitive to different depths) following Yanovskaya & Ditmar^60^. The Rayleigh wave tomography maps are then inverted to retrieve the 3D S-wave velocity structure in the second step. The estimation of S-wave velocity from the dispersion data is a non-linear geophysical inverse problem. Traditionally, the inversion is linearized and is solved for velocity with a fixed number of layers. Additionally, a damping parameter is used to stabilize the inversion^24^. Therefore, proper quantification of uncertainties becomes challenging. Here, we apply a fully non-linear Bayesian approach^25,26,61^, which does not require any damping, to compute the 1D shear velocity profiles and their uncertainties. The parameter uncertainties can either come from measurement errors or from theoretical errors.

In Bayesian inversion, the answer to the inverse problem is expressed in terms of posterior probability density (PPD), which combines the prior information (what we know beforehand about the model and is independent of data) and the likelihood (incorporates the data information), i.e., $p({\boldsymbol{m}}/{\boldsymbol{d}})\propto p({\boldsymbol{d}}/{\boldsymbol{m}})p({\boldsymbol{m}})$. Here, *p*(***m***/***d***) is the probability of the model parameter vector (***m***) given the data vector (***d***) (i.e. posterior probability density), *p*(***d***/***m***) is the probability of the data given the model (i.e., likelihood) and *p*(***m***) is the prior probability of the model parameters (i.e., number of layers, layer thickness and S velocity). The data errors are typically not known and are approximated as a difference between the measured and predicted data. Note that the P-wave velocity is derived from the fixed Vp/Vs ratio and density is derived from S-wave velocity. In this paper, we consider a uniform prior within a range of reasonable S-wave velocity (based on the past studies) as a function of depth while the likelihood function is derived based on a Gaussian distribution of data errors.

It is challenging to compute the posterior analytically, particularly for non-linear inversion. Additionally, the model complexity (i.e., the number of layers in the case of observed data) is not known in advance and estimated parameter uncertainties can highly depend on the model complexity. For example, if we increase the model complexity (i.e., increase the number of layers), the fit between the model prediction and observed data can be improved, but not necessarily required by data and can result in unrealistically large uncertainties. In contrast, a simple model can fit only part of the data resulting in unreasonably small uncertainties. Therefore, a parameter sampling approach known as reversible jump Markov chain Monte Carlo (rjMcMC) sampling is applied to compute the PPD^26,62^.

In rjMcMC approach, the number of layers is allowed to change (between 1 and 35 from the surface to 100 km depth) and parameters in each iteration are updated through three different moves: birth, death, and perturbation. In the case of a birth move, a new interface at random depth is introduced and proposed with the perturbation of velocity and thickness from a randomly chosen layer. The proposed model is accepted or rejected based on the likelihood ratio of the current model to the previous model. If the proposal is accepted, the model is updated with an additional layer and proceeds for the next iteration. If the proposal is rejected then the current model is retained and a new model is proposed again. In the case of death move, a random layer is picked and proposed to delete (death) with the perturbation of layer thickness and velocity from a randomly chosen layer. Then the same procedure as in the case of the birth stage is followed. In the case of perturbation move, the number of layers remains the same and only layer properties (layer thickness and velocity) are allowed to change.

The sampling approach, particularly rjMcMC can be highly inefficient when significantly low probability regions separate multiple high probability regions. As a result, the sampling takes a long time to converge to the true model. Therefore, interacting Markov chains are applied here to achieve faster convergence. For more details, we refer to Dettmer & Dosso^25^ and Pachhai *et al*.^61^.

## Data Availability

The time series data are available on the Orfeus website (https://www.orfeus-eu.org/). The trans-dimensional code can be provided by S. Pachhai (spachhai@ucsd.edu) on request.

## Supplementary information

Supplementary material
