# Author Correction: 3D shear wave velocity model of the crust and uppermost mantle beneath the Tyrrhenian basin and margins

**DOI:** 10.1038/s41598-019-48083-7

**Published:** 2019-08-15

**Authors:** D. Manu-Marfo, A. Aoudia, S. Pachhai, R. Kherchouche

**Affiliations:** 1 The Abdus Salam International Center for Theoretical Physics, Trieste, Italy; 2 University of Trieste, Trieste, Italy; 3 Bullard Laboratories, Department of Earth Sciences, University of Cambridge, Cambridge, UK; 4 Now at Institute of Geophysics and Planetary Physics, Scripps Institution of Oceanography, University of California, San Diego, California USA; 5 Université des Sciences et de la Technologie Houari Boumediene, Algiers, Algeria; 6 Now at Centre de Recherche en Astronomie, Astrophysique et Géophysique, Algiers, Algeria

Correction to: *Scientific Reports* 10.1038/s41598-019-40510-z, published online 05 March 2019

The original version of this Article omitted an affiliation for author Radia Kherchouche. The correct affiliations are listed below.

1- The Abdus Salam International Center for Theoretical Physics, Trieste, Italy;

5- Université des Sciences et de la Technologie Houari Boumediene, Algiers, Algeria;

6- Now at Centre de Recherche en Astronomie, Astrophysique et Géophysique, Algiers, Algeria.

This has now been corrected in the HTML and PDF versions of this Article, and in the accompanying Supplemental Material.

