# Supplementary material for: 3D shear wave velocity model of the crust and uppermost mantle beneath the Tyrrhenian basin and margins
Source: Sci Rep. 2019 Mar 5;9:3609. doi: 10.1038/s41598-019-40510-z (PMC6401166; doi:10.1038/s41598-019-40510-z)
Supplement: Supplementary file 1 — Supplementary material [file 41598_2019_40510_MOESM1_ESM.docx]

**3D shear wave velocity model of the crust and uppermost mantle beneath the Tyrrhenian basin and margins**

**D. Manu-Marfo^1,2^, A. Aoudia^1,^**^*^**, S. Pachhai^1,3,4^, & R. Kherchouche ^1,5,6^**

^1^The Abdus Salam International Center for Theoretical Physics, Trieste, Italy.

^2^University of Trieste, Trieste, Italy.

^3^Bullard Laboratories, Department of Earth Sciences, University of Cambridge, Cambridge, UK.

^4^Now at Institute of Geophysics and Planetary Physics, Scripps Institution of Oceanography, University of California, San Diego, California, USA.

^5^Université des Sciences et de la Technologie Houari Boumediene, Algiers, Algeria.

^6^Now at Centre de Recherche en Astronomie, Astrophysique et Géophysique, Algiers, Algeria.

* Corresponding author: A. Aoudia ([aoudia@ictp.it](mailto:aoudia@ictp.it))

**Supplementary Information**

**Figures S1 – S10**

**Moho depth estimation**

**Depth resolution tests**

**Inversion of observed measurements**

**References**


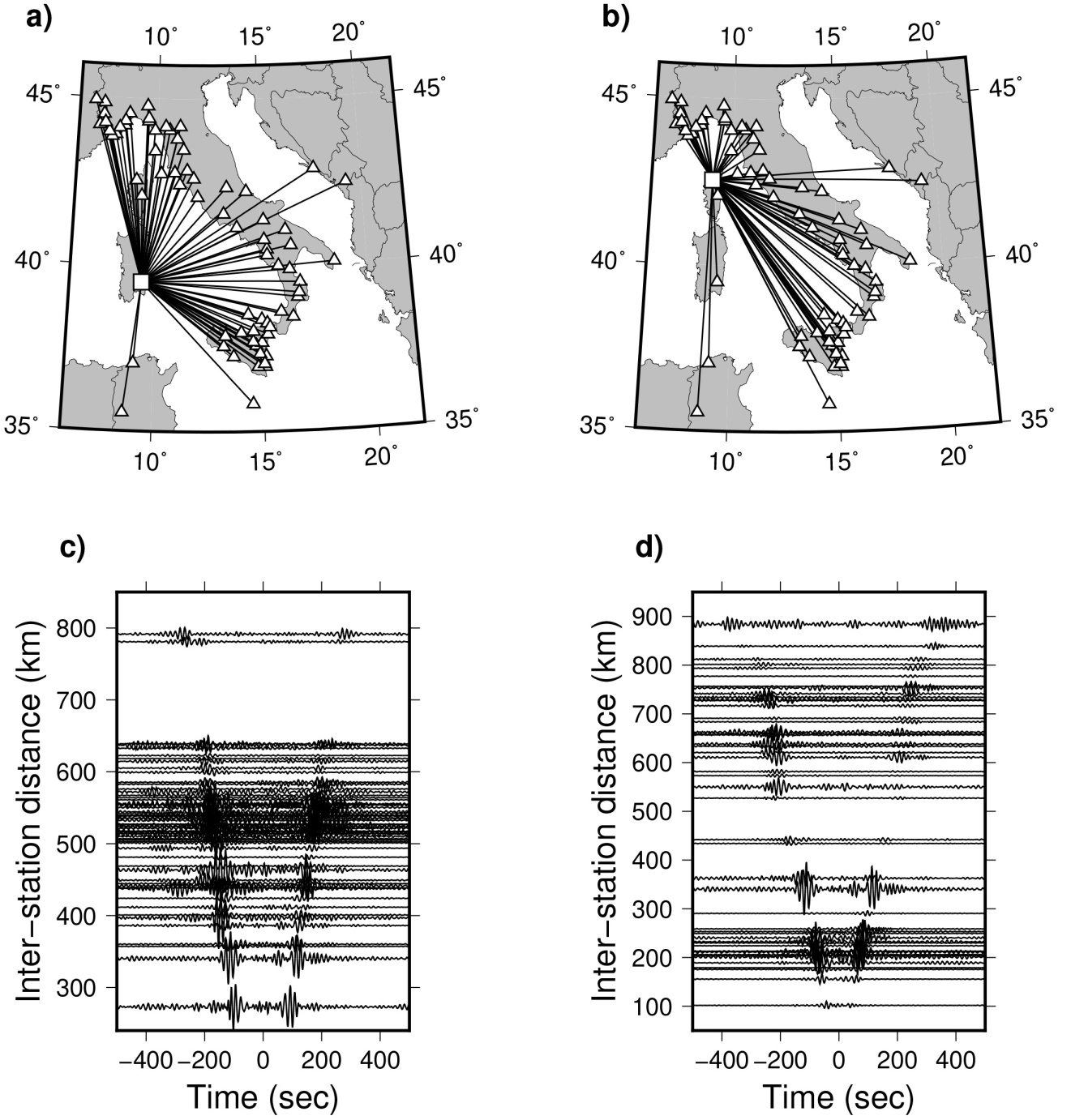


**Figure S1**. Inter-station ray-path between (a) center station VSL and all other stations, (b) center station PGT2 and the rest of the stations. (c-d) Examples of record section of cross-correlation calculated for the ray-paths in a and b, respectively. The cross-correlation functions have been bandpass filtered between 5 – 15 s.


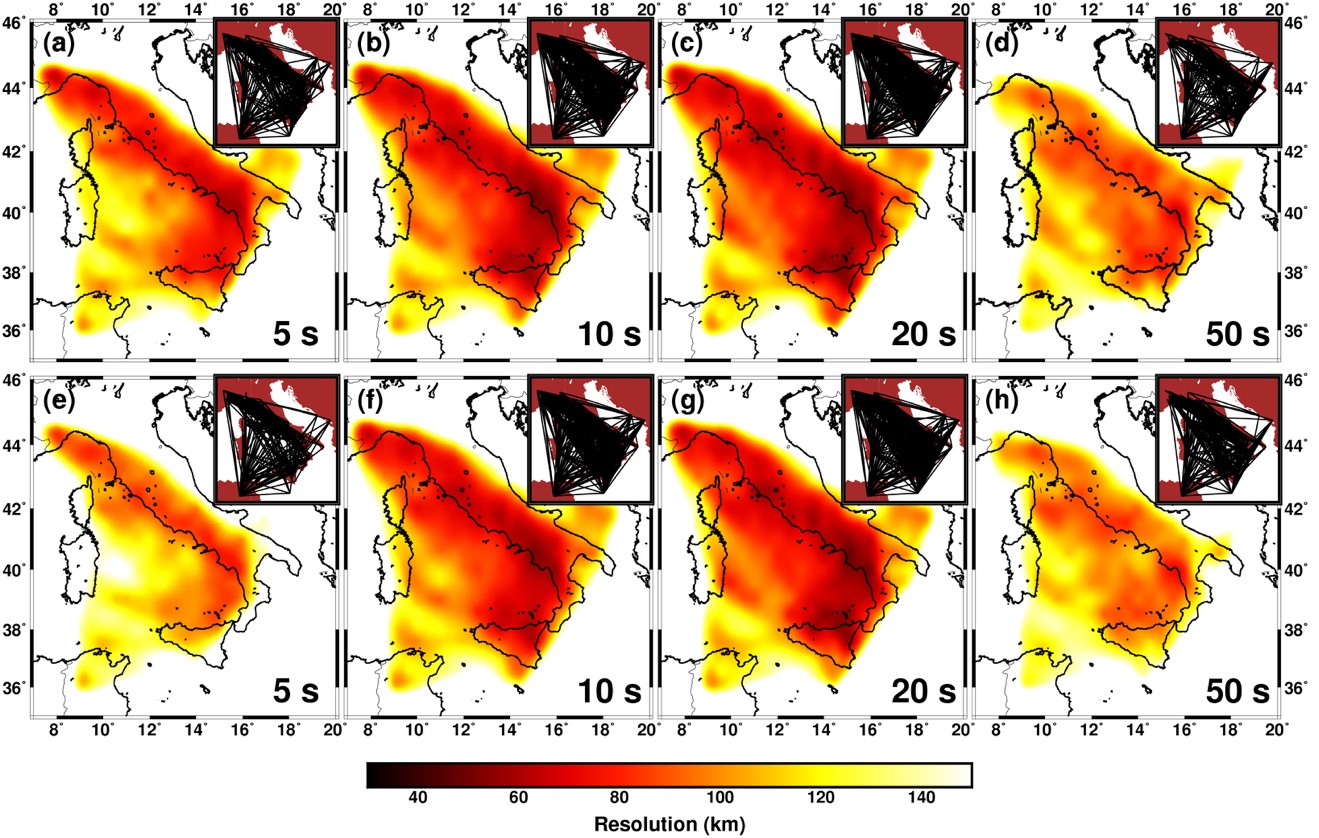


**Figure S2**. The estimated resolution map obtained from the surface wave tomography method. Resolution estimates for (a-d) group velocity and (e-h) phase velocity at 5, 10, 20 and 50 s, respectively. The azimuthal paths coverage used for tomography inversion at each period is shown on the inset map.


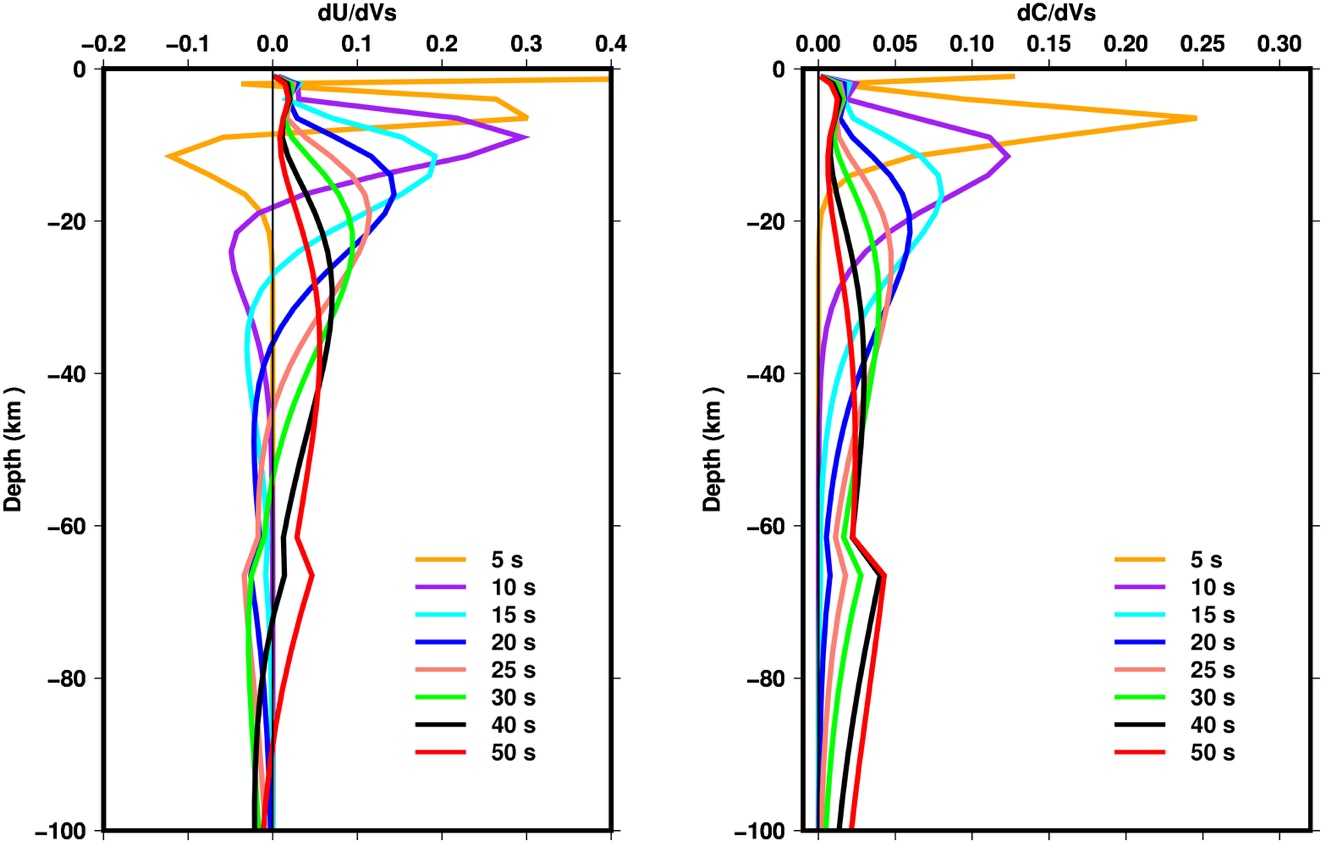


**Figure S3**. Sensitivity kernels for Rayleigh-wave (left) group and (right) phase velocity for different periods.


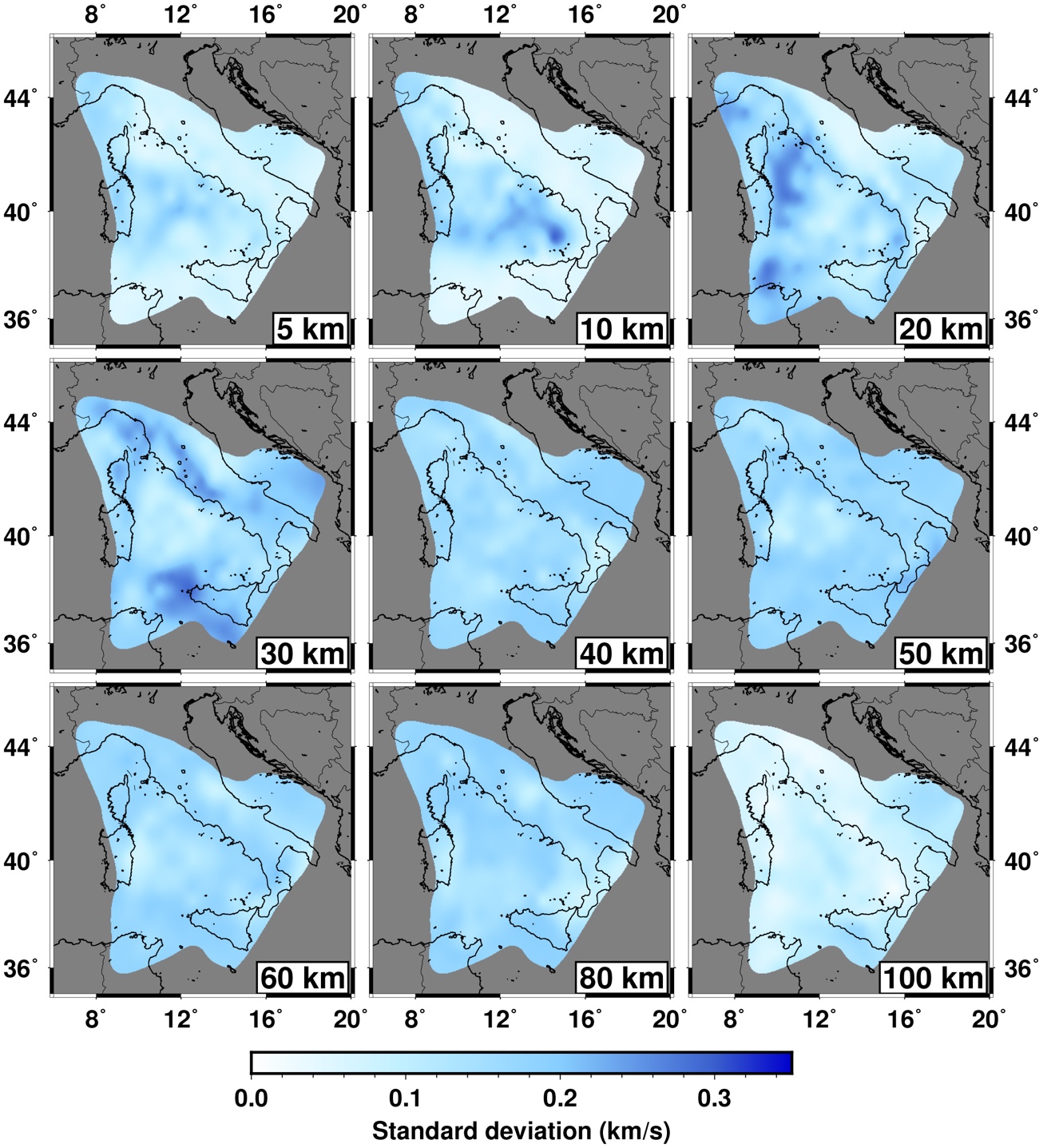


**Figure S4**. Horizontal slices of standard deviations of the 3-D shear velocity model.


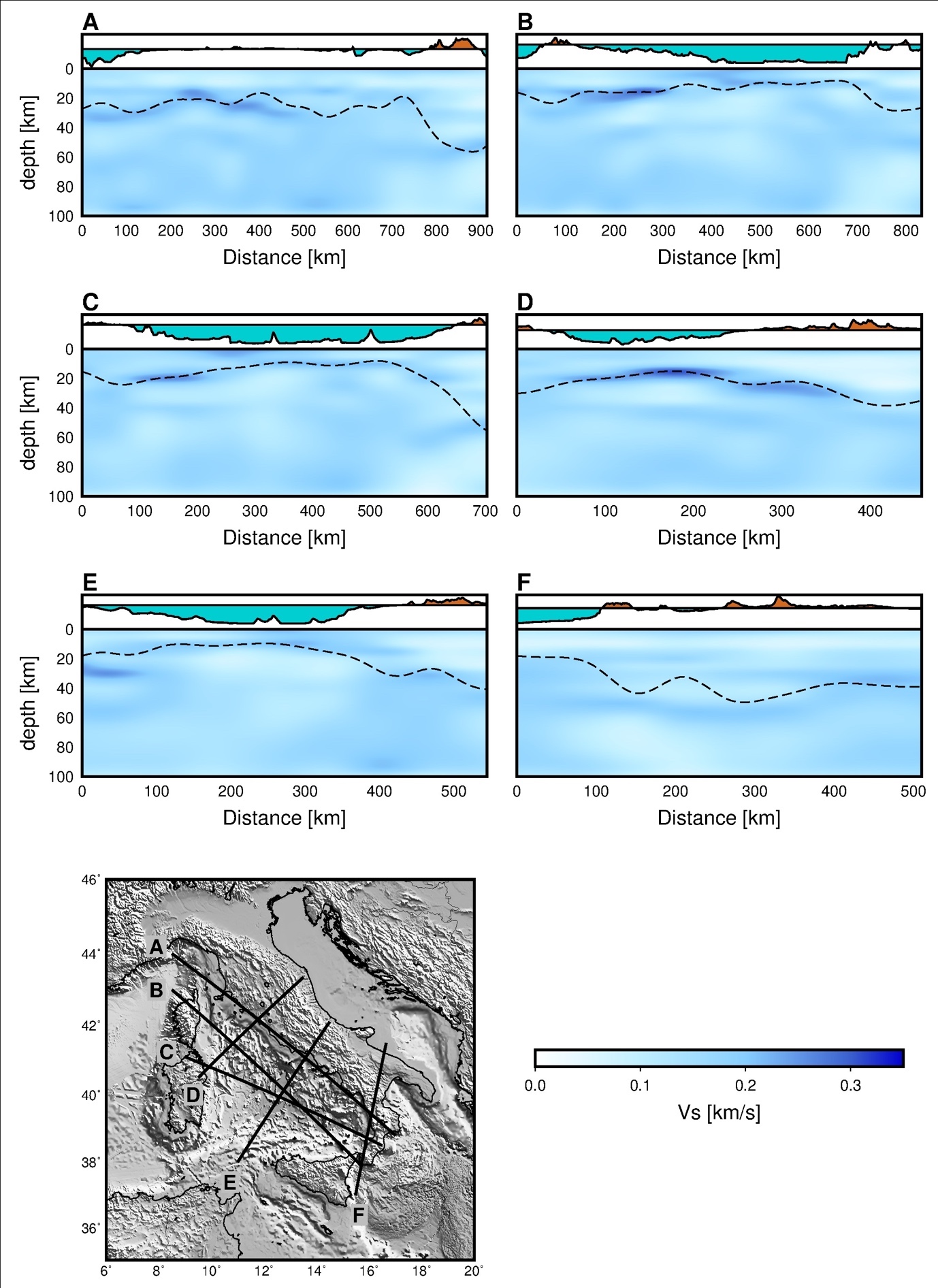


**Figure S5**. Vertical sections of the standard deviations of the 3-D shear velocity model along profiles shown in Fig. 4. The dashed line shows the Moho undulation along the profile.


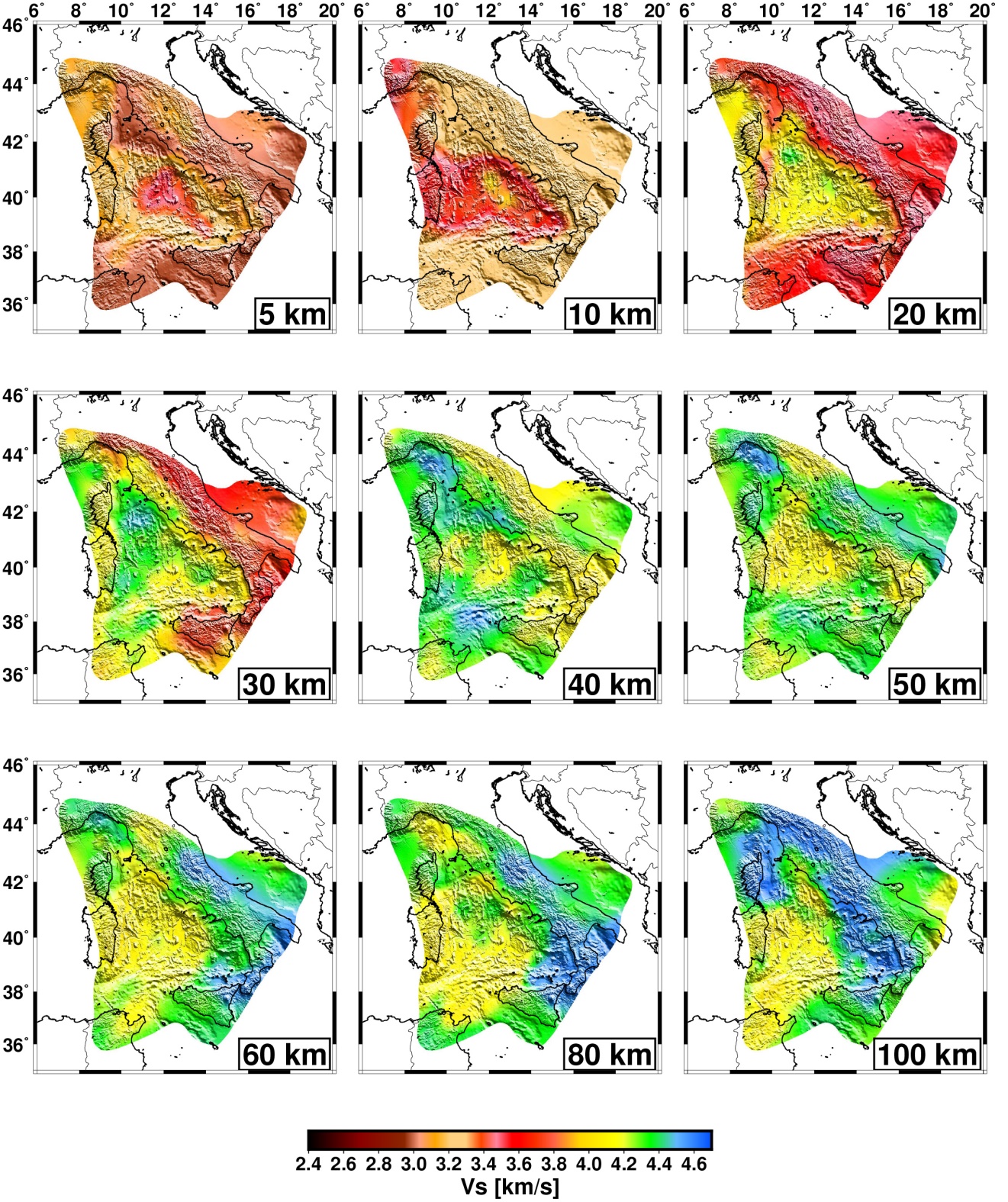


**Figure S6**. Shear velocity model at different depths beneath the Tyrrhenian basin.


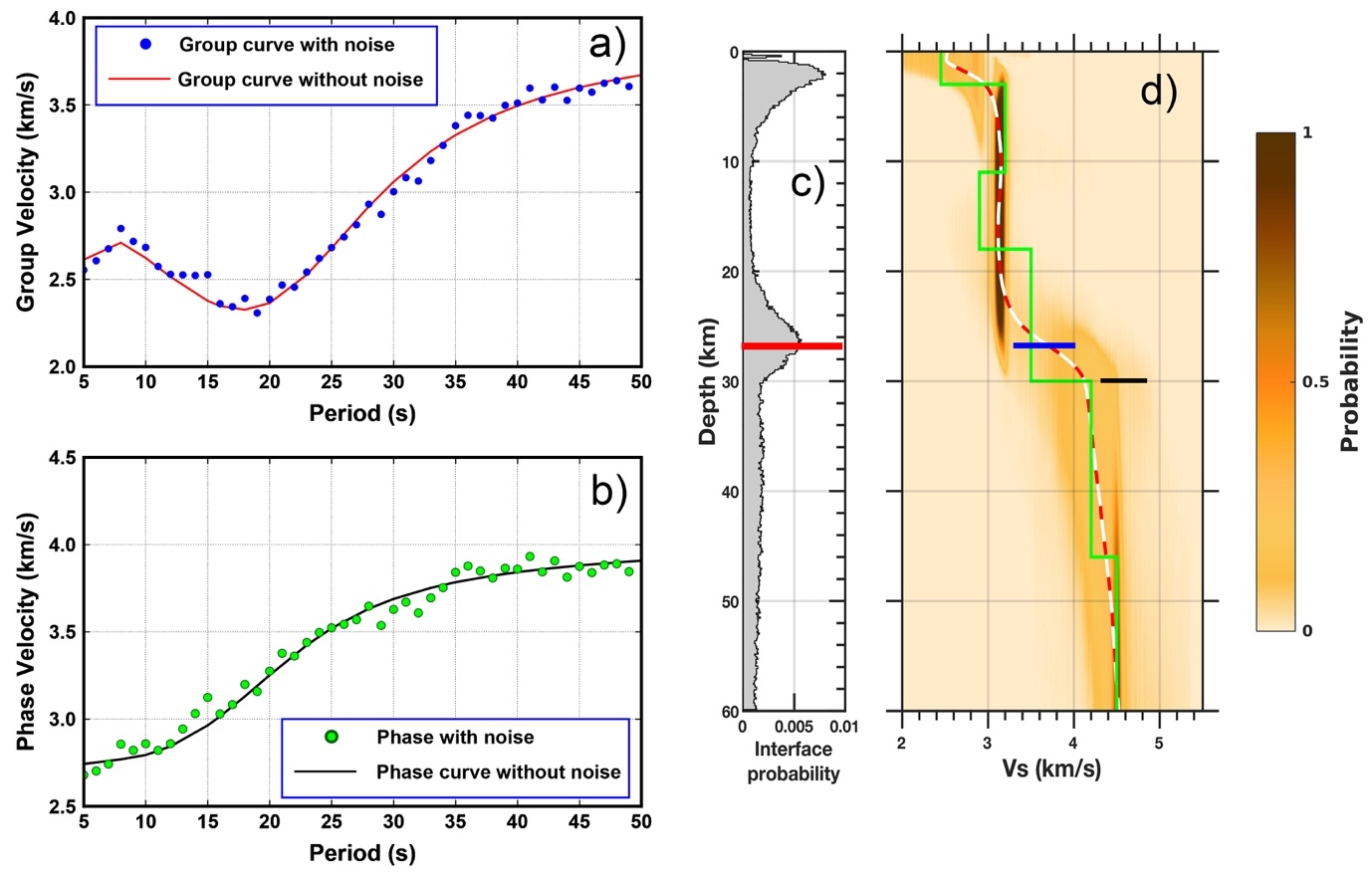


**Figure S7**. Trans-dimensional Bayesian inversion results for synthetic dispersion curves. Synthetic dispersion curves for (a) group velocity and (b) phase velocity with and without noise. (c) Posterior probability for the position of discontinuities. The horizontal red line marks the location of retrieved Moho discontinuity. (d) Posterior Probability Density (PPD) for shear velocity as a function of depth. The green solid line indicates the true 1-D velocity model used to prepare data in a and b. Similarly, the dashed white and red line represent the smooth mean model retrieved from the inversion. We use the depth difference between the blue and the black lines on (d) to estimate the uncertainty of the Moho depth.

**
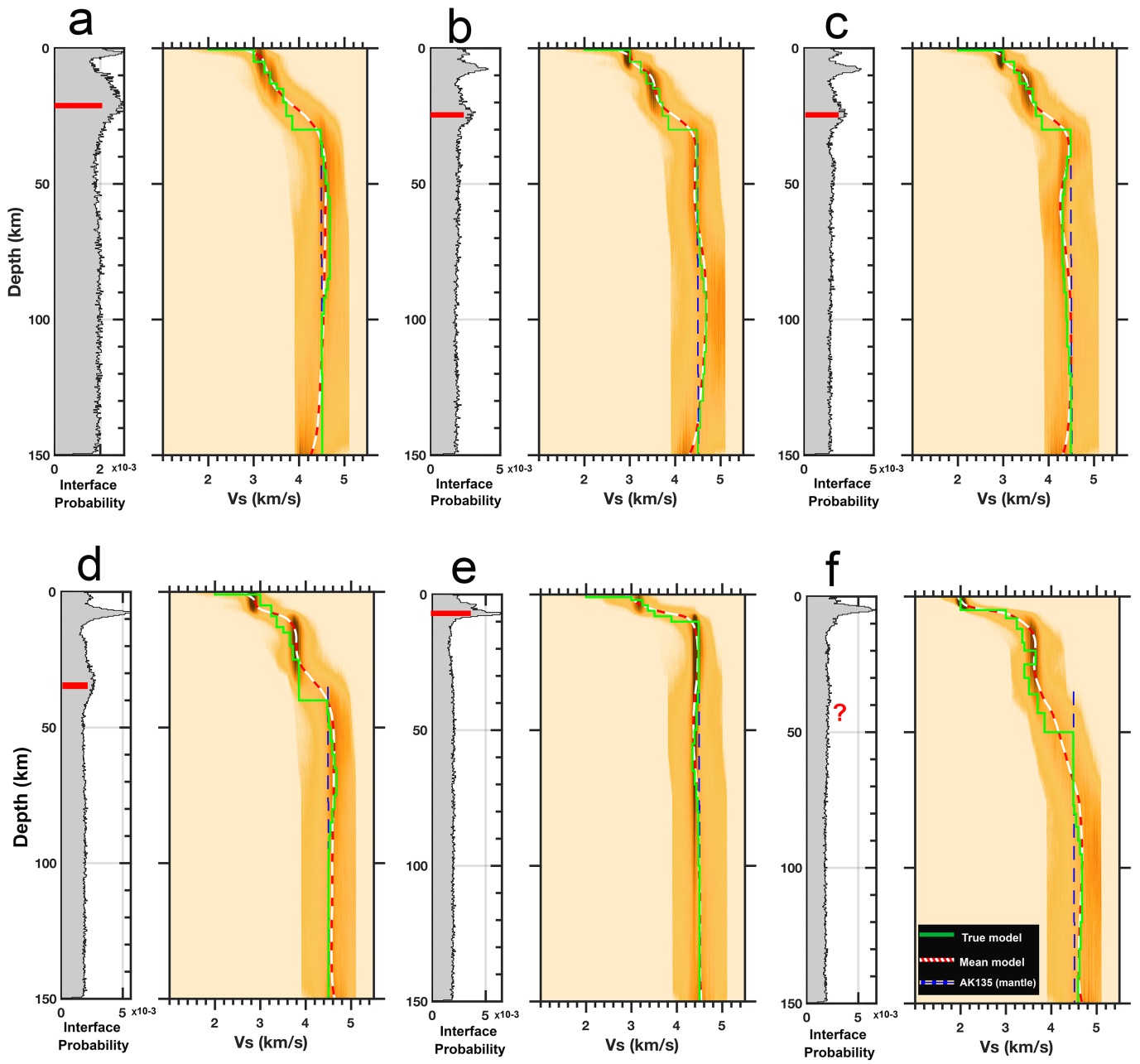
**

**Figure S8**. Tests showing the depth resolution of the applied inversion method. Here we compare six different true models (green line) to the recovered models (red and white line). Dashed blue lines show the AK135 mantle velocity. The Moho depth is estimated from the interface probability along each velocity depth profile.


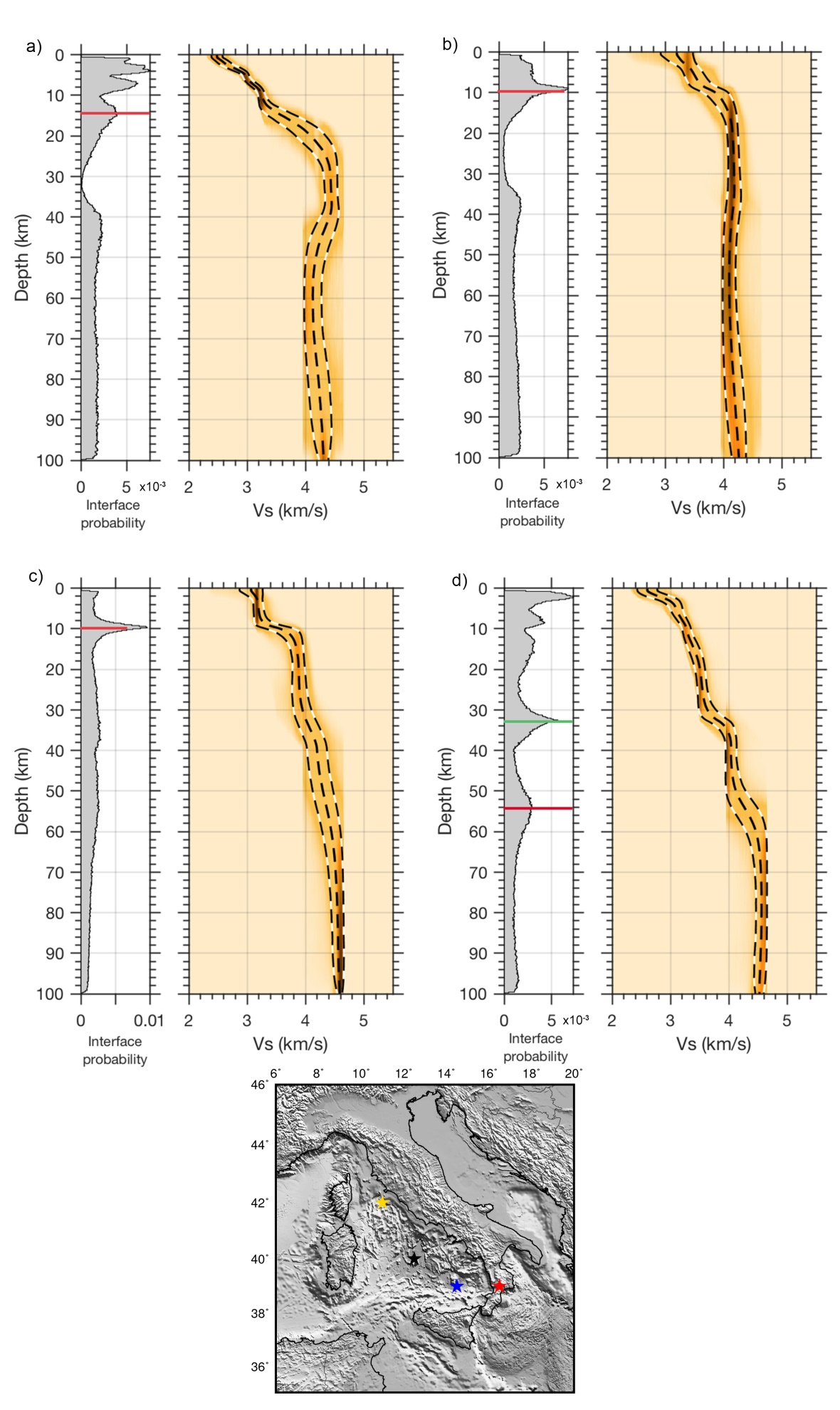


**Figure S9**. Shear-velocity structure for grid nodes in (a) the northern Tyrrhenian basin (yellow star on map), (b) the Vavilov basin (black star on map), (c) Marsili basin (blue star on map) and (d) Calabria (red star on map). The horizontal red line depicts the depth of the Moho discontinuity estimated in this study. The horizontal green line on (d) indicates a possible double Moho in agreement with previous studies from receiver functions^1^.

**
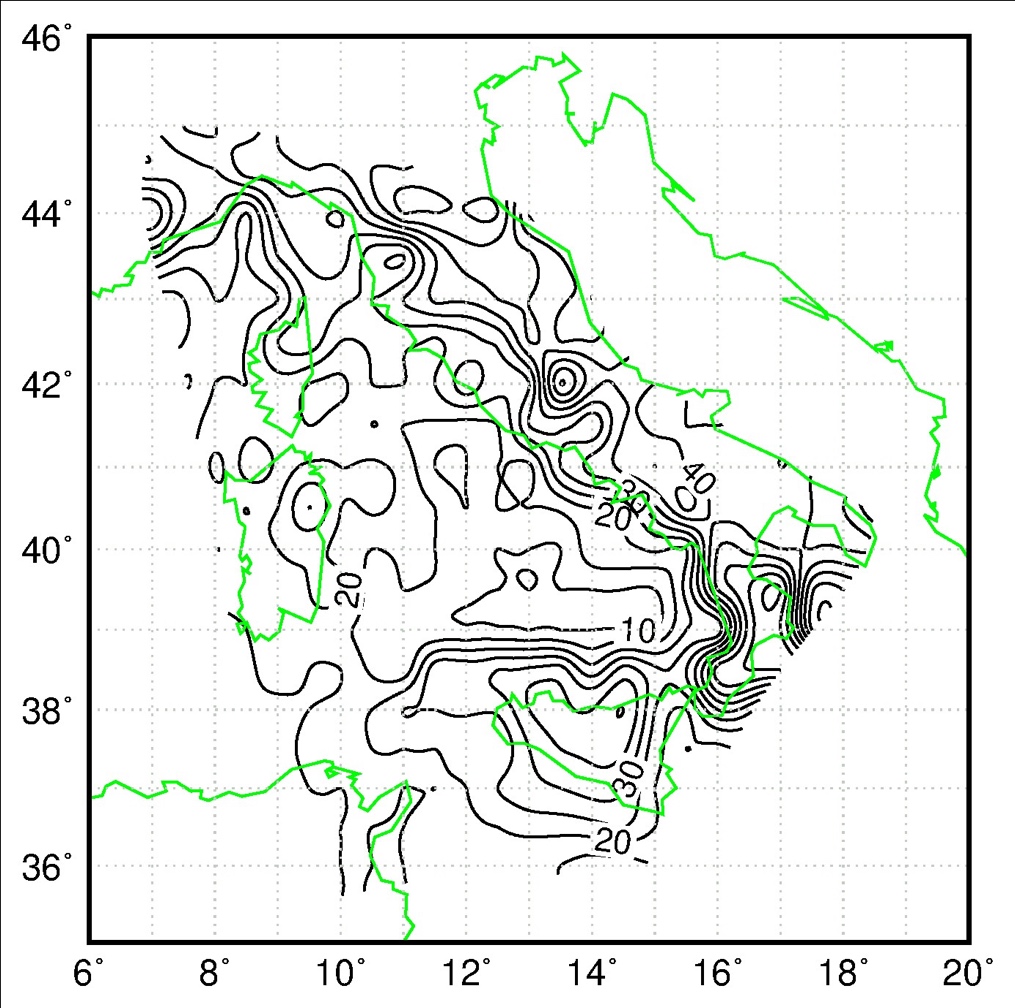
**

**Figure S10**. The contour of the Moho depth topography estimated in this study. The Moho depths found here are very consistent with crustal thickness found in previous studies^1–3^.

Moho depth estimation

We estimate the Moho depth topography for the study area by analyzing the probabilistic 1-D shear velocity-depth profile at each grid node for the depth where there is a probability for a discontinuity at a pertinent shear-wave velocity. The pertinent shear-wave velocity corresponds to velocities > 3.6 km/s, considering that the average shear velocity of the crust is ~3.5 km/s. Surface wave dispersion measurements are generally sensitive to absolute shear wave velocities but are poor in constraining discontinuities. Here, we test the feasibility and accuracy of using the joint inversion of group and phase dispersion measurements applying the trans-dimensional Bayesian inversion method to constrain the Moho discontinuity from the interface probability. For this experiment, we first compute the group and phase velocity dispersions for 1-D velocity model with a Moho at 30 km. Then a correlated noise is added to the synthetic data to make representative observed data (Fig. S7a and b). These data are then inverted for the shear velocity profile using the trans-dimensional Bayesian inversion method. The first one-third of samples are discarded and the average of the remaining samples are considered to visualize probabilities of discontinuity and S velocity as a function of depths. The inversion results (Fig. S7c and d) show that the absolute velocities from the true model are well constrained by the average shear velocity (red and white dash line in Fig. S7d), and two strong discontinuities (out of the five from the true model) are recovered at ~2.5 km and at ~27 km (Fig. S7c). We consider the second discontinuity at ~27 km to correspond to the Moho interface, which is set at 30 km in the true model (Fig. S7d). For all the test we carried out, we observe that the recovered Moho interface is underestimated. This may be because of the poor sensitivity of the dispersion data to the location of discontinuities. As a result, the velocity discontinuity smears out giving maximum probability near the average velocity between layers (e.g., Figs. S7 and S8). Again, we notice that the mean shear velocity at the Moho depth (blue line, Fig. S7d) does not correspond to the velocity of the underlying mantle but rather an average of the crust and mantle velocities. This sometimes causes the Moho depth when plotted on the velocity sections as in Figure 4, in some places, crosses low velocity structures. We estimate the uncertainty associated with the Moho depth by obtaining the difference in depth between the recovered Moho (blue line, Fig. S7d) and the depth at the start of the mantle velocity (black line, Fig. S7d) which usually is where the gradient of the mean velocity is approximately infinite. When applied to real data as shown in Figure S9, the Moho depth from the interface probability is in good agreement with inferred Moho depths from previous studies.

**Depth Resolution Tests**

We test the resolution capabilities of the trans-dimensional Bayesian inversion by creating Rayleigh wave group and phase synthetic dispersion curves for different models with different Moho depth and variable crustal complexities. Figure S8 shows the true models used to compute the synthetics and the inversion results. All the true models have crustal velocity structure sandwiched between a sedimentary basin and underlying mantle structure following AK135 apart from one smooth velocity anomaly. A correlated noise is added to the synthetic dispersion curves.

The inversion is most sensitive to the shallow structure given the narrow distribution of the velocities from the ensemble models and uncertainty increases as a function of depth. The inversion is unable to constrain the velocity of the shallow sedimentary layers. This is expected since our surface wave dispersion curves do not include periods shorter than 5 s. Interestingly, for most of the tests shown in Figure S8, the interface probability shows the presence of a shallow layer with a thickness less or equal to 1 km likely corresponding to the sedimentary layer. The presence of this shallow interface may be the result of the large impedance contrast between the sedimentary layer and the crust. When the sedimentary layer is 5 km thick (Fig. S8f), inversion constrains both the absolute velocity and the location of the discontinuity very well, suggesting that very thick sedimentary basin can be resolved very well.

We estimate the depth of the Moho from the interface probability (Fig. S8). There are some discrepancies between the recovered and true Moho locations when we use maximum interface probability approach. In all cases, Moho depth is understimated. The differences are ~2 km for the shallow Moho (Fig. S8e) and ~5 km for deeper Moho (Figs S8b-d). For very complex crustal structure shown in Figure S8f, the interface probability is unable to uniquely resolve the Moho. In such a case, we estimate the Moho as the depth where the strongest velocity gradient occurs at a pertinent velocity. In the case where we strongly perturb the input dispersion curves (Fig. S8a), the Moho depth shows an error of ~10 km. The consistency of the interface probability to provide an estimate of the Moho depth even for models with slightly weak impendence contrast (not shown here) suggest that it is a viable way to estimate the Moho depth from the 1-D profiles. However, we have to be cautious about the inherent problem of using surface waves to locate discontinuities which can cause wider uncertainties on the Moho depth location.

The inversion is able to retrieve all the mantle anomalies down to 150 km in their tendency (positive/negative velocity anomaly with respect to AK135) although the peak amplitude is sometimes underestimated or overestimated. Deeper (Figs S8b and f ) as well as narrower (Figs S8d and e) anomalies are well estimated by the inversion. The inversion underestimates the anomaly in Figure S8a but recovers the velocity above the anomaly very well.

**Inversion of observed measurements**

Figure S9 shows how the inversion fared on real data. We show four velocity profiles from the Bayesian inversion using local dispersion curves extracted from group and phase tomography maps. Figure S9a is the inversion results for a grid point in the northern Tyrrhenian basin, where the Moho depth is ~16 km^4^. In the Vavilov and Marsili basin, we observe a possible Moho depth at ~10 km (Figs S9b and c, respectively). The velocity-depth profile beneath Calabria (Fig. S9d) indicates two probably interfaces at ~33 and 54 km, given hints about the possibility of a double Moho in agreement with Receiver function results^1^. Figure S10 shows the contour map of the Moho topography estimated for the Tyrrhenian basin and margins from the interface probability. This map agrees with the most recent published map of the Moho topography of the study area^2^.

References

1. Piana Agostinetti, N. & Amato, A. Moho depth and Vp / Vs ratio in peninsular Italy from teleseismic receiver functions. *J. Geophys. Res.* **114,** B06303 (2009).

2. Spada, M., Bianchi, I., Kissling, E., Agostinetti, N. P. & Wiemer, S. Combining controlled-source seismology and receiver function information to derive 3-D moho topography for Italy. *Geophys. J. Int.* **194,** 1050–1068 (2013).

3. Di Stefano, R., Bianchi, I., Ciaccio, M. G., Carrara, G. & Kissling, E. Three-dimensional Moho topography in Italy: New constraints from receiver functions and controlled source seismology. *Geochemistry, Geophys. Geosystems* **12,** 1–15 (2011).

4. Moeller, S. *et al.* Early-stage rifting of the northern Tyrrhenian Sea Basin: Results from a combined wide-angle and multichannel seismic study. *Geochemistry, Geophys. Geosystems* **14,** 3032–3052 (2013).
